# Supplementary figures and images for: celldeath: A tool for detection of cell death in transmitted light microscopy images by deep learning-based visual recognition
Source: PLoS One. 2021 Jun 24;16(6):e0253666. doi: 10.1371/journal.pone.0253666 (PMC8224851; doi:10.1371/journal.pone.0253666)

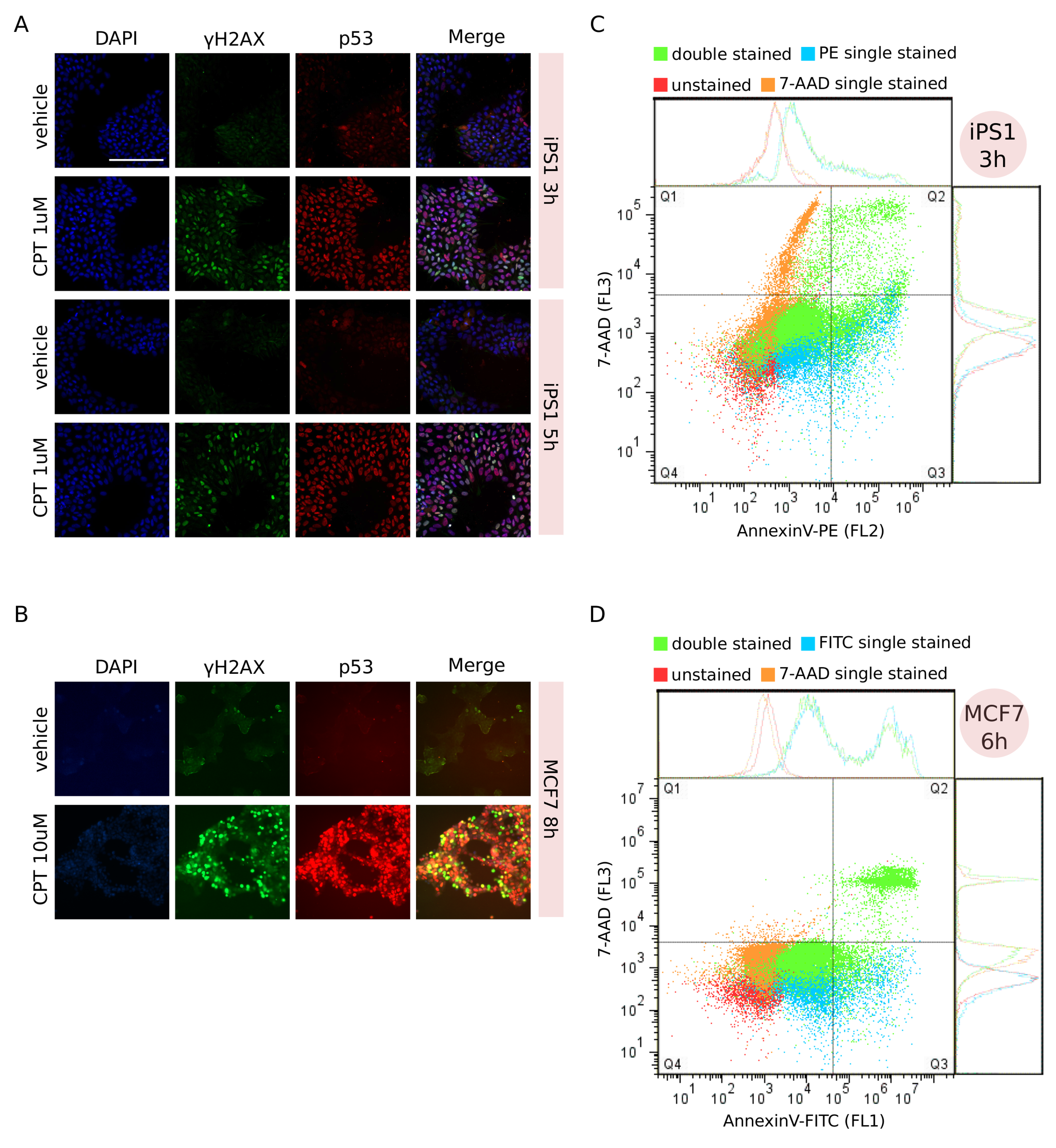

Supplement: S1 Fig — A) iPS1 cells were treated or not (DMSO) with CPT 1uM for 3 and 5h. Cells were stained with anti-γH2AX or anti-p53 and nuclei were revealed with DAPI. Scale was set to 200um (white bar). B) MCF7 cells were treated or not (DMSO) with CPT 10uM for 8h. Cells were stained as in A. C) Controls used for setting background levels in iPS1 flow cytometry experiments. D) Controls used for setting background levels in MCF7 flow cytometry experiments. (TIFF) [file pone.0253666.s004.tiff]

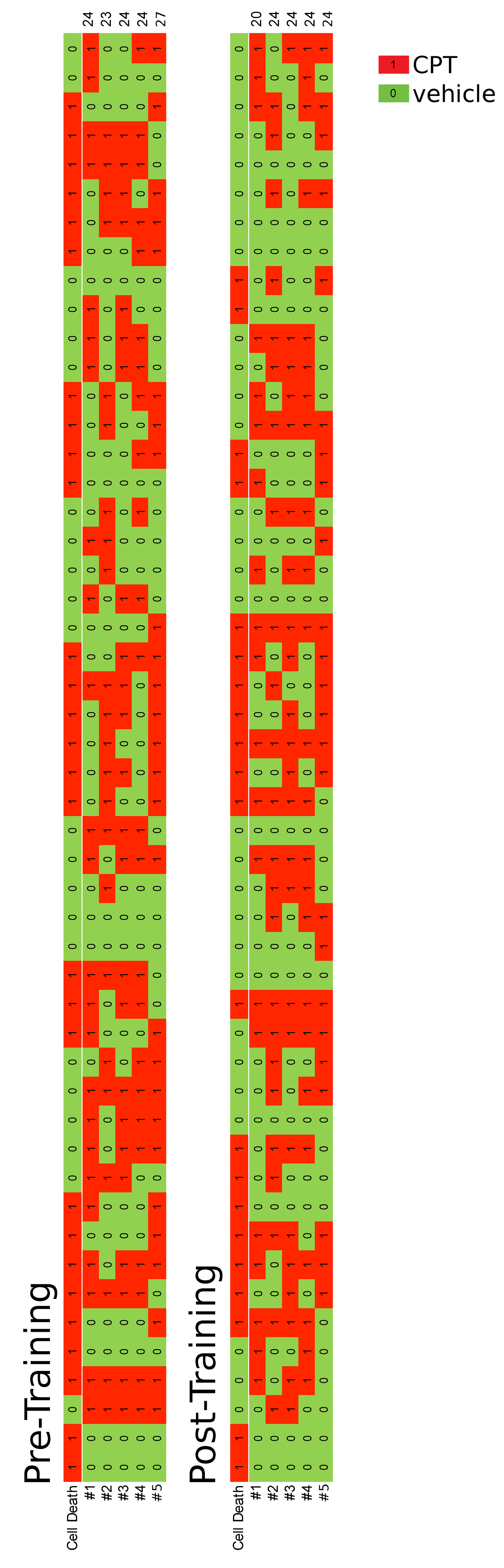

Supplement: S2 Fig — Detailed results of five subjects involved in scientific activities tested for their capacity to discriminate cells treated with CPT from DMSO before (Pre-) and after (Post-) being trained with a different set of images. (TIFF) [file pone.0253666.s005.tiff]

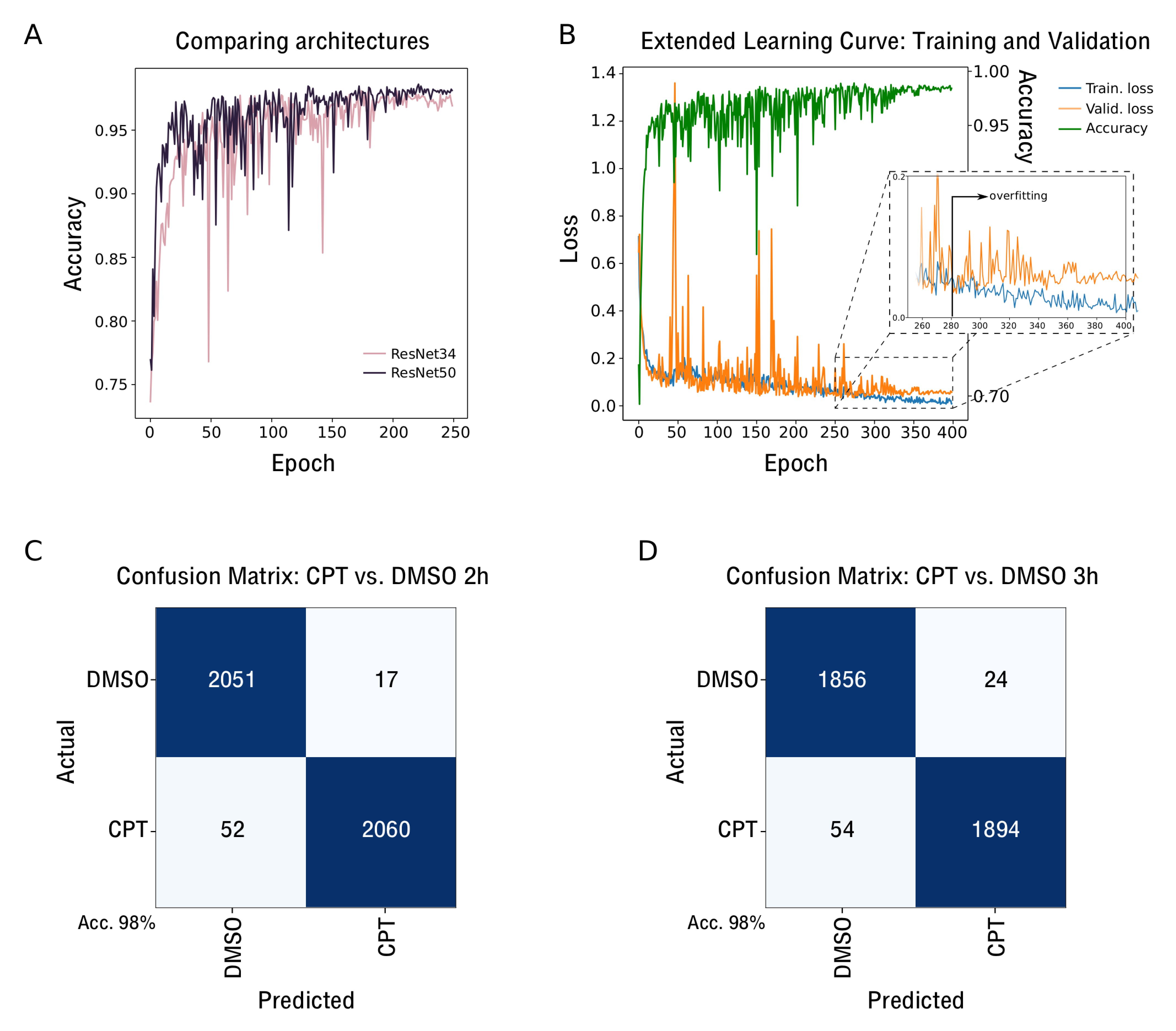

Supplement: S3 Fig — A) Comparison of accuracy results between ResNet50 and ResNet34 architectures using the same input data and parameters. B) Learning curve (training and validation sets) for ResNet50 architecture during extended training (400 epochs). Point of inflection in validation curve is indicated with an arrow inside the inset box. Validation accuracy for the training run is also shown. C) Confusion matrix for images of 2h CPT/DMSO-treated cells. D) Confusion matrix for images of 3h CPT/DMSO-treated cells. (TIFF) [file pone.0253666.s006.tiff]
