# Supplementary material for: celldeath: A tool for detection of cell death in transmitted light microscopy images by deep learning-based visual recognition
Source: PLoS One. 2021 Jun 24;16(6):e0253666. doi: 10.1371/journal.pone.0253666 (PMC8224851; doi:10.1371/journal.pone.0253666)
Supplement: Supplementary file 5 [file pone.0253666.s002.odt]

S2 Table. Deep learning model specifications

Sequential

======================================================================

Layer (type)         Output Shape         Param #    Trainable

======================================================================

Conv2d               [64, 240, 320]       9,408      False     

______________________________________________________________________

BatchNorm2d          [64, 240, 320]       128        True      

______________________________________________________________________

ReLU                 [64, 240, 320]       0          False     

______________________________________________________________________

MaxPool2d            [64, 120, 160]       0          False     

______________________________________________________________________

Conv2d               [64, 120, 160]       4,096      False     

______________________________________________________________________

BatchNorm2d          [64, 120, 160]       128        True      

______________________________________________________________________

Conv2d               [64, 120, 160]       36,864     False     

______________________________________________________________________

BatchNorm2d          [64, 120, 160]       128        True      

______________________________________________________________________

Conv2d               [256, 120, 160]      16,384     False     

______________________________________________________________________

BatchNorm2d          [256, 120, 160]      512        True      

______________________________________________________________________

ReLU                 [256, 120, 160]      0          False     

______________________________________________________________________

Conv2d               [256, 120, 160]      16,384     False     

______________________________________________________________________

BatchNorm2d          [256, 120, 160]      512        True      

______________________________________________________________________

Conv2d               [64, 120, 160]       16,384     False     

______________________________________________________________________

BatchNorm2d          [64, 120, 160]       128        True      

______________________________________________________________________

Conv2d               [64, 120, 160]       36,864     False     

______________________________________________________________________

BatchNorm2d          [64, 120, 160]       128        True      

______________________________________________________________________

Conv2d               [256, 120, 160]      16,384     False     

______________________________________________________________________

BatchNorm2d          [256, 120, 160]      512        True      

______________________________________________________________________

ReLU                 [256, 120, 160]      0          False     

______________________________________________________________________

Conv2d               [64, 120, 160]       16,384     False     

______________________________________________________________________

BatchNorm2d          [64, 120, 160]       128        True      

______________________________________________________________________

Conv2d               [64, 120, 160]       36,864     False     

______________________________________________________________________

BatchNorm2d          [64, 120, 160]       128        True      

______________________________________________________________________

Conv2d               [256, 120, 160]      16,384     False     

______________________________________________________________________

BatchNorm2d          [256, 120, 160]      512        True      

______________________________________________________________________

ReLU                 [256, 120, 160]      0          False     

______________________________________________________________________

Conv2d               [128, 120, 160]      32,768     False     

______________________________________________________________________

BatchNorm2d          [128, 120, 160]      256        True      

______________________________________________________________________

Conv2d               [128, 60, 80]        147,456    False     

______________________________________________________________________

BatchNorm2d          [128, 60, 80]        256        True      

______________________________________________________________________

Conv2d               [512, 60, 80]        65,536     False     

______________________________________________________________________

BatchNorm2d          [512, 60, 80]        1,024      True      

______________________________________________________________________

ReLU                 [512, 60, 80]        0          False     

______________________________________________________________________

Conv2d               [512, 60, 80]        131,072    False     

______________________________________________________________________

BatchNorm2d          [512, 60, 80]        1,024      True      

______________________________________________________________________

Conv2d               [128, 60, 80]        65,536     False     

______________________________________________________________________

BatchNorm2d          [128, 60, 80]        256        True      

______________________________________________________________________

Conv2d               [128, 60, 80]        147,456    False     

______________________________________________________________________

BatchNorm2d          [128, 60, 80]        256        True      

______________________________________________________________________

Conv2d               [512, 60, 80]        65,536     False     

______________________________________________________________________

BatchNorm2d          [512, 60, 80]        1,024      True      

______________________________________________________________________

ReLU                 [512, 60, 80]        0          False     

______________________________________________________________________

Conv2d               [128, 60, 80]        65,536     False     

______________________________________________________________________

BatchNorm2d          [128, 60, 80]        256        True      

______________________________________________________________________

Conv2d               [128, 60, 80]        147,456    False     

______________________________________________________________________

BatchNorm2d          [128, 60, 80]        256        True      

______________________________________________________________________

Conv2d               [512, 60, 80]        65,536     False     

______________________________________________________________________

BatchNorm2d          [512, 60, 80]        1,024      True      

______________________________________________________________________

ReLU                 [512, 60, 80]        0          False     

______________________________________________________________________

Conv2d               [128, 60, 80]        65,536     False     

______________________________________________________________________

BatchNorm2d          [128, 60, 80]        256        True      

______________________________________________________________________

Conv2d               [128, 60, 80]        147,456    False     

______________________________________________________________________

BatchNorm2d          [128, 60, 80]        256        True      

______________________________________________________________________

Conv2d               [512, 60, 80]        65,536     False     

______________________________________________________________________

BatchNorm2d          [512, 60, 80]        1,024      True      

______________________________________________________________________

ReLU                 [512, 60, 80]        0          False     

______________________________________________________________________

Conv2d               [256, 60, 80]        131,072    False     

______________________________________________________________________

BatchNorm2d          [256, 60, 80]        512        True      

______________________________________________________________________

Conv2d               [256, 30, 40]        589,824    False     

______________________________________________________________________

BatchNorm2d          [256, 30, 40]        512        True      

______________________________________________________________________

Conv2d               [1024, 30, 40]       262,144    False     

______________________________________________________________________

BatchNorm2d          [1024, 30, 40]       2,048      True      

______________________________________________________________________

ReLU                 [1024, 30, 40]       0          False     

______________________________________________________________________

Conv2d               [1024, 30, 40]       524,288    False     

______________________________________________________________________

BatchNorm2d          [1024, 30, 40]       2,048      True      

______________________________________________________________________

Conv2d               [256, 30, 40]        262,144    False     

______________________________________________________________________

BatchNorm2d          [256, 30, 40]        512        True      

______________________________________________________________________

Conv2d               [256, 30, 40]        589,824    False     

______________________________________________________________________

BatchNorm2d          [256, 30, 40]        512        True      

______________________________________________________________________

Conv2d               [1024, 30, 40]       262,144    False     

______________________________________________________________________

BatchNorm2d          [1024, 30, 40]       2,048      True      

______________________________________________________________________

ReLU                 [1024, 30, 40]       0          False     

______________________________________________________________________

Conv2d               [256, 30, 40]        262,144    False     

______________________________________________________________________

BatchNorm2d          [256, 30, 40]        512        True      

______________________________________________________________________

Conv2d               [256, 30, 40]        589,824    False     

______________________________________________________________________

BatchNorm2d          [256, 30, 40]        512        True      

______________________________________________________________________

Conv2d               [1024, 30, 40]       262,144    False     

______________________________________________________________________

BatchNorm2d          [1024, 30, 40]       2,048      True      

______________________________________________________________________

ReLU                 [1024, 30, 40]       0          False     

______________________________________________________________________

Conv2d               [256, 30, 40]        262,144    False     

______________________________________________________________________

BatchNorm2d          [256, 30, 40]        512        True      

______________________________________________________________________

Conv2d               [256, 30, 40]        589,824    False     

______________________________________________________________________

BatchNorm2d          [256, 30, 40]        512        True      

______________________________________________________________________

Conv2d               [1024, 30, 40]       262,144    False     

______________________________________________________________________

BatchNorm2d          [1024, 30, 40]       2,048      True      

______________________________________________________________________

ReLU                 [1024, 30, 40]       0          False     

______________________________________________________________________

Conv2d               [256, 30, 40]        262,144    False     

______________________________________________________________________

BatchNorm2d          [256, 30, 40]        512        True      

______________________________________________________________________

Conv2d               [256, 30, 40]        589,824    False     

______________________________________________________________________

BatchNorm2d          [256, 30, 40]        512        True      

______________________________________________________________________

Conv2d               [1024, 30, 40]       262,144    False     

______________________________________________________________________

BatchNorm2d          [1024, 30, 40]       2,048      True      

______________________________________________________________________

ReLU                 [1024, 30, 40]       0          False     

______________________________________________________________________

Conv2d               [256, 30, 40]        262,144    False     

______________________________________________________________________

BatchNorm2d          [256, 30, 40]        512        True      

______________________________________________________________________

Conv2d               [256, 30, 40]        589,824    False     

______________________________________________________________________

BatchNorm2d          [256, 30, 40]        512        True      

______________________________________________________________________

Conv2d               [1024, 30, 40]       262,144    False     

______________________________________________________________________

BatchNorm2d          [1024, 30, 40]       2,048      True      

______________________________________________________________________

ReLU                 [1024, 30, 40]       0          False     

______________________________________________________________________

Conv2d               [512, 30, 40]        524,288    False     

______________________________________________________________________

BatchNorm2d          [512, 30, 40]        1,024      True      

______________________________________________________________________

Conv2d               [512, 15, 20]        2,359,296  False     

______________________________________________________________________

BatchNorm2d          [512, 15, 20]        1,024      True      

______________________________________________________________________

Conv2d               [2048, 15, 20]       1,048,576  False     

______________________________________________________________________

BatchNorm2d          [2048, 15, 20]       4,096      True      

______________________________________________________________________

ReLU                 [2048, 15, 20]       0          False     

______________________________________________________________________

Conv2d               [2048, 15, 20]       2,097,152  False     

______________________________________________________________________

BatchNorm2d          [2048, 15, 20]       4,096      True      

______________________________________________________________________

Conv2d               [512, 15, 20]        1,048,576  False     

______________________________________________________________________

BatchNorm2d          [512, 15, 20]        1,024      True      

______________________________________________________________________

Conv2d               [512, 15, 20]        2,359,296  False     

______________________________________________________________________

BatchNorm2d          [512, 15, 20]        1,024      True      

______________________________________________________________________

Conv2d               [2048, 15, 20]       1,048,576  False     

______________________________________________________________________

BatchNorm2d          [2048, 15, 20]       4,096      True      

______________________________________________________________________

ReLU                 [2048, 15, 20]       0          False     

______________________________________________________________________

Conv2d               [512, 15, 20]        1,048,576  False     

______________________________________________________________________

BatchNorm2d          [512, 15, 20]        1,024      True      

______________________________________________________________________

Conv2d               [512, 15, 20]        2,359,296  False     

______________________________________________________________________

BatchNorm2d          [512, 15, 20]        1,024      True      

______________________________________________________________________

Conv2d               [2048, 15, 20]       1,048,576  False     

______________________________________________________________________

BatchNorm2d          [2048, 15, 20]       4,096      True      

______________________________________________________________________

ReLU                 [2048, 15, 20]       0          False     

______________________________________________________________________

AdaptiveAvgPool2d    [2048, 1, 1]         0          False     

______________________________________________________________________

AdaptiveMaxPool2d    [2048, 1, 1]         0          False     

______________________________________________________________________

Flatten              [4096]               0          False     

______________________________________________________________________

BatchNorm1d          [4096]               8,192      True      

______________________________________________________________________

Dropout              [4096]               0          False     

______________________________________________________________________

Linear               [512]                2,097,664  True      

______________________________________________________________________

ReLU                 [512]                0          False     

______________________________________________________________________

BatchNorm1d          [512]                1,024      True      

______________________________________________________________________

Dropout              [512]                0          False     

______________________________________________________________________

Linear               [2]                  1,026      True      

______________________________________________________________________

Total params: 25,615,938

Total trainable params: 2,161,026

Total non-trainable params: 23,454,912

Optimized with 'torch.optim.adam.Adam', betas=(0.9, 0.99)

Using true weight decay as discussed in [https://www.fast.ai/2018/07/02/adam-weight-decay/](https://www.fast.ai/2018/07/02/adam-weight-decay/)

Loss function : FlattenedLoss

======================================================================

Callbacks functions applied
