# Supplementary material for: celldeath: A tool for detection of cell death in transmitted light microscopy images by deep learning-based visual recognition
Source: PLoS One. 2021 Jun 24;16(6):e0253666. doi: 10.1371/journal.pone.0253666 (PMC8224851; doi:10.1371/journal.pone.0253666)
Supplement: Attachment — Submitted filename: Response to Reviewers.pdf [file pone.0253666.s007.pdf]

Santiago Miriuka, MD MSc PhD FACC
LIAN
Fleni Institute-CONICET
Email: smiriuka@fleni.org.ar

RE: Revised submission PONE-D-21-04850

May 20, 2021

celldeath: a tool for detection of cell death in transmitted light microscopy images by deep learning-based visual recognition

Alejandro La Greca [a,‡], Nelba Pérez [a,‡], Sheila Castañeda [a], Paula Melania Milone,[a], María Agustina Scarafía [a], Alan Miqueas Möbbs [a], Ariel Waisman [a], Lucía Moro [a], Gustavo Sevlever [a], Carlos Luzzani [a], Santiago Gabriel Miriuka [a,*]

[a]LIAN-CONICET, FLENI, Buenos Aires, Argentina. ‡ These authors contributed equally to this work
* Corresponding author: smiriuka@fleni.org.ar

Short title: ”Cell death detection by CNN”

Dear Dr. Chi-Hua Chen,

Hereby we submit a revised version of our manuscript entitled “celldeath: a tool for detection of cell death in transmitted light microscopy images by deep learning-based visual recognition” by Alejandro La Greca and collaborators. We have addressed all concerns and requirements indicated by the Journal, as well as comments raised by Reviewer 1 in a point-by-point fashion.

Modifications introduced to the manuscript are properly declared in the“Response to Reviewers” letter and they are clearly marked in the “Revised Manuscript with Track Changes” pdf file. Also, a revised unmarked “Manuscript” pdf file has been included with this submission along with the LaTex source file.

Journal Requirements

1. **Please ensure that your manuscript meets PLOS ONE’s style requirements, including those for file naming.**
   As a result of using PLOS ONE’s latex template, our manuscript follows all necessary style requirements.
2. **Please review your reference list to ensure that it is complete and correct.**
   Reference list was properly checked.
3. **PLOS requires an ORCID iD for the corresponding author in Editorial Manager on papers submitted after December 6th, 2016.**
   We have associated the ORCID iD of Dr. Santiago Miriuka (corresponding author) to this submission.
4. **In your Data Availability statement, you have not specified where the minimal data set underlying the results described in your manuscript can be found (only ”image data”).**
   The minimal data set to reproduce all CNN trainings and tests can be found in https://www.kaggle.com/miriukalaboratory/cell-death-in-seven-cell-lines.
5. **Please amend the manuscript submission data (via Edit Submission) to include author Paula Melania Milone.**
   Manuscript submission data has been amended to include author Paula Melania Milone.
6. **Please remove any funding-related text from the manuscript and let us know how you would like to update your Funding Statement.**
   Funding-related text has been removed from manuscript. Funding Statement should go as follows:
   This work was supported by grants to Dr. Miriuka from the National Scientific and Technical Research Council (CONICET) PIP112-20150100723 and from the Scientific and Technical Research Fund (FONCyT) PICT2016-0544.

Reviewers' comments

Reviewer #1

We are grateful for Reviewer #1's comments and insightful remarks. Issues raised by Reviewer have been addressed below.

The authors should provide a more direct visual comparison between accepted measures of cell death (i.e., fluorescence based) and their network output. Fig 4 provides a very crude visualization of this, but it is impossible to tell how real the output is without a direct comparison to a ground truth.

We believe that Reviewer #1 raised a reasonable point considering that we proposed that our model markedly outperforms humans and could eventually replace laborious, expensive or repetitive experimental procedures. While it is true that Fig4 could further explore the features regarded as relevant to the network, we did not attempt to construct an explanatory model from our trained CNN (see Shmueli et al. 2010 for further details on this matter). In fact, it would be inaccurate to decode features responsible for classification through predictive models -such as neural networks-. As a consequence, the signal observed in the class activation maps will not necessarily correlate with any fluorescent mark. At present, however, state-of-the-art computer-vision (field of image recognition) algorithms are being developed in an attempt to better interpret "what is the network actually seeing" and perhaps reduce model biases and generalization errors (Guidotti et al. 2019). Our intention with Fig4 was simply to demonstrate that our model was extracting features potentially important for classification from cell-occupied regions of the images rather than background.

We have included an explicit reference to this issue in revised manuscript (Discussion, paragraph 2).

The authors should provide a more meaningful discussion regarding how these results may (or may not) be extrapolated. Will a network need to be retrained for every cell type under every different condition? Or will this approach enable a more universal assessment of cell death after training with representative cells in perhaps non-identical circumstances?

In agreement with Reviewer's suggestion, we have included a more significant discussion on this topic in the revised manuscript (Discussion, paragraph 5). Also, we revised the corresponding text in Results section to provide clearer statements (Results, subsection "CNN identifies very early features of cell death", paragraph 3).

Regards,

Santiago Miriuka, MD MSc PhD FACC
